# Assessing the evolution of scientific publications in orthopedics journals from mainland China, Hong Kong, and Taiwan: a 12-year survey of the literature

**DOI:** 10.1186/s13018-016-0404-z

**Published:** 2016-06-17

**Authors:** Hua Jiang, Bingjin Nong, Lijing Yang, Shaohui Zong, Xinli Zhan, Qingjun Wei, Zengming Xiao

**Affiliations:** Department of Orthopedic Surgery, The First Affiliated Hospital of Guangxi Medical University, Shuangyong Road No. 6, Nanning, 530021 China; Library of Medicine, Guangxi Medical University, Nanning, China

**Keywords:** China, Medical publication, Orthopedics, Research

## Abstract

**Background:**

In China, the field of orthopedics has experienced significant growth over the past 12 years. However, the recent status of research on orthopedics among individuals in mainland China, Hong Kong, and Taiwan is unknown. In this study, we investigated characteristics and trends of orthopedics publications from these three regions.

**Methods:**

Between 2003 and 2014, all articles published in 63 orthopedics journals originating from mainland China, Hong Kong, and Taiwan were identified via Science Citation Index Expanded (SCIE) database. A survey was conducted to systematically analyze the published orthopedics articles from the three regions according to the numbers of articles, study design, impact factors (IFs), citations, most prolific authors, and institutions. Additionally, we evaluated global trends in orthopedics publications, and ranked top 10 countries in terms of the total number of published articles over 12 years and the number of published articles per year.

**Results:**

A total number of 123,317 articles were published in the 63 orthopedics journals between 2003 and 2014. The worldwide number of annually published orthopedics articles tended to increase during the study period. The total number of orthopedics publications from the three regions, especially in mainland China, increased markedly from 2003 to 2014. The annual number of orthopedics articles from mainland China increased from 6 in 2003 to 813 in 2014, Hong Kong increased from 32 in 2003 to 71 in 2014, and Taiwan increased from 68 in 2003 to 168 in 2014. For accumulated IFs and total citations of articles, mainland China ranked the first place, followed by Taiwan and Hong Kong. However, publications from Taiwan had the highest average citations per article, and publications from Hong Kong had the highest average IFs. Among the top 10 most prolific authors and institutions, 4 authors and 4 institutions were from Taiwan, 3 authors and 4 institutions were from mainland China, and 3 authors and 2 institutions were from Hong Kong.

**Conclusions:**

The quantity of articles published in international orthopedics journals from mainland China presented a remarkable upward trend during the past 12 years. Given the relative size of the populations, it should be emphasized that mainland China still has a long way to go to achieve the academic performance of Hong Kong and Taiwan.

## Background

Over the past 30 years, China has scored impressive economic and cultural achievements since the adoption of the reform and opening up policy. With rapid socioeconomic development of China, great changes have taken place in science and medicine during the past decade [1]. The number of scientific papers published annually from China ranked second in the world after 2006 [2]. During the same period, Chinese orthopedics research productivity has flourished, and has got more international attention [3].

Bibliometric analyses provide significant advantages for the identification of the quantity and quality of publications from a specific country. These insights may be helpful to benchmark our scientific output and aid the allocation of future research funding [4]. Thus, bibliometrics to measure scientific productivity has been more notable in recent years. Self-assessment of the orthopedics publication from individual countries has been reported by Turkey and Japan [5, 6]. However, information regarding Chinese scientific contribution in the field of orthopedics is still lacking. The purpose of this study is to investigate the evolution of orthopedics studies in mainland China, Hong Kong, and Taiwan, by analyzing the trends and characteristics of the scientific articles published in orthopedics journals.

## Methods

In the present study, a total of 65 orthopedics journals were selected from the Science Citation Index (SCI) and Science Citation Index Expanded (SCIE) database. The selection criteria, as previous studies described [7], included that the journal met all of the following requirements: (1) was listed in the “orthopedics” category of SCI or SCIE for 2014; (2) was indexed in the PubMed database; and (3) had impact factors (IFs) according to Thomson Reuters Journal Citation Reports (JCR) 2014. *The Bone & Joint Journal*, which did not have an IF in JCR 2014, and *Osteologie*, which was not indexed by PubMed, were excluded from this study. A computerized literature search was conducted using the “PubMed” and “Web of Knowledge” databases on September 1st, 2015, and the articles published from January 1st 2003 to December 31st 2014 in these journals were retrieved. The orthopedics articles from mainland China, Hong Kong, and Taiwan were identified using the first author’s institutional affiliations. The full journal titles or the ISSN numbers of the journals were used to perform this search. The search terms used were “0363-5465 OR 1063-4584 OR 0021-9355 OR 0031-9023 OR 0749-8063 OR 0736-0266 OR 1836-9553 OR 0009-921X OR 0942-2056 OR 0301-620X OR 1529-9430 OR 0940-6719 OR 0020-1383 OR 1745-3674 OR 0362-2436 OR 1067-151X OR 0190-6011 OR 0883-5403 OR 1058-2746 OR 0966-6362 OR 1753-1934 OR 0341-2695 OR 1050-642X OR 0300-8207 OR 1471-2474 OR 1536-0652 OR 0268-0033 OR 1757-1146 OR 0894-1130 OR 0968-0160 OR 0030-5898 OR 0363-5023 OR 1071-1007 OR 1749-799X OR 0890-5339 OR 0091-3847 OR 0271-6798 OR 0936-8051 OR 1877-0568 OR 0309-3646 OR 0749-0712 OR 1053-8127 OR 0949-2658 OR 1413-3555 OR 1067-2516 OR 0147-7447 OR 1083-7515 OR 1120-7000 OR 0085-4530 OR 1060-152X OR 1305-8282 OR 0019-5413 OR 1864-6697 OR 0744-6020 OR 8750-7315 OR 0934-6694 OR 0001-6462 OR 1017-995X OR 2000-656X OR 0973-6042 OR 0891-8422 OR 0001-5415 OR 0959-3020 OR 0932-0555 OR 1413-7852” AND “Hong Kong [ad],” “Taiwan [ad],” and “China [ad] NOT Hong Kong [ad] NOT Taiwan [ad].” Publication type of clinical studies were classified into clinical trials, randomized controlled trials (RCTs), and case reports, and were calculated using data obtained from the PubMed.

Systematic analyses have been performed on included orthopedics articles. First, we acquired and compiled the information of scientific literature from the three regions of China based on the JCR 2014 [8], which contained accumulated and average IFs of publications, citations of articles, publication type of clinical research, distribution of contributing regions. Then, we analyzed these data from the three regions of China. Second, we quantified the articles published in top 10 orthopedics journals with the highest IFs, and selected the top 10 popular orthopedics journals for the three regions in terms of the number of published articles, and ranked the most prolific authors/institutions according to the number of articles and citations. Moreover, we calculated the total and annual numbers of published orthopedics articles worldwide, and ranked top 10 countries in terms of the total number of published articles over 12 years and the number of published articles per year. Two reviewers (HJ and BN) independently extracted the articles. When disagreement existed between the two reviewers, a third reviewer (ZX) was consulted to resolve.

### Statistical analyses

Statistical analyses were performed using SPSS 20.0 software (IBM, New York, USA), and the statistical results are shown as Tables and Figures. The Kruskal-Wallis test and the Mann-Whitney test were used to detect differences between regions. The trends with respect to the number of articles were analyzed via curvilinear regression. Significance was tested using the two-tailed test, and *P* < 0.05 was considered significant.

## Results

### Global trends in orthopedics publications

A total number of 123,317 articles were published in the 63 orthopedics journals from 2003 to 2014. The worldwide number of annually published orthopedics articles tended to increase during the study period. The United States of America (USA) ranked the highest in the number of published orthopedics articles (36,893 articles), followed by the UK (6886), Japan (5867), China (5521), Germany (4356), Canada (3732), South Korea (2939), the Netherlands (2432), Australia (2355), and Turkey (2019). Based on the number of published articles per year, top 10 countries were described in Table 1.

**Table 1:** Top 10 countries according to the annual number of articles from 2003 to 2014

Year	1	2	3	4	5	6	7	8	9	10
2003	USA	United Kingdom	Japan	Germany	Canada	France	Australia	Switzerland	Netherlands	Turkey
2004	USA	United Kingdom	Japan	Germany	Canada	France	Australia	Switzerland	Turkey	Netherlands
2005	USA	United Kingdom	Japan	Germany	Canada	France	Australia	Netherlands	Turkey	Switzerland
2006	USA	United Kingdom	Japan	Germany	Canada	France	Switzerland	Sweden	Australia	Netherlands
2007	USA	United Kingdom	Japan	Germany	Canada	France	Australia	Netherlands	South Korea	Turkey
2008	USA	United Kingdom	Japan	Germany	Canada	France	Turkey	South Korea	Netherlands	Australia
2009	USA	United Kingdom	Japan	Germany	Canada	France	South Korea	Turkey	Netherlands	China
2010	USA	United Kingdom	Japan	Germany	Canada	Netherlands	France	Australia	China	South Korea
2011	USA	United Kingdom	Japan	Germany	China	Canada	South Korea	Australia	France	Switzerland
2012	USA	United Kingdom	China	Japan	Germany	South Korea	Canada	Netherlands	Australia	Italy
2013	USA	United Kingdom	China	Japan	Germany	South Korea	Canada	Netherlands	Australia	Switzerland
2014	USA	United Kingdom	China	Japan	Germany	Canada	South Korea	Netherlands	Italy	Australia

*Abbreviations*: *USA* United States of America

### Total number of articles

**Fig. 1:**
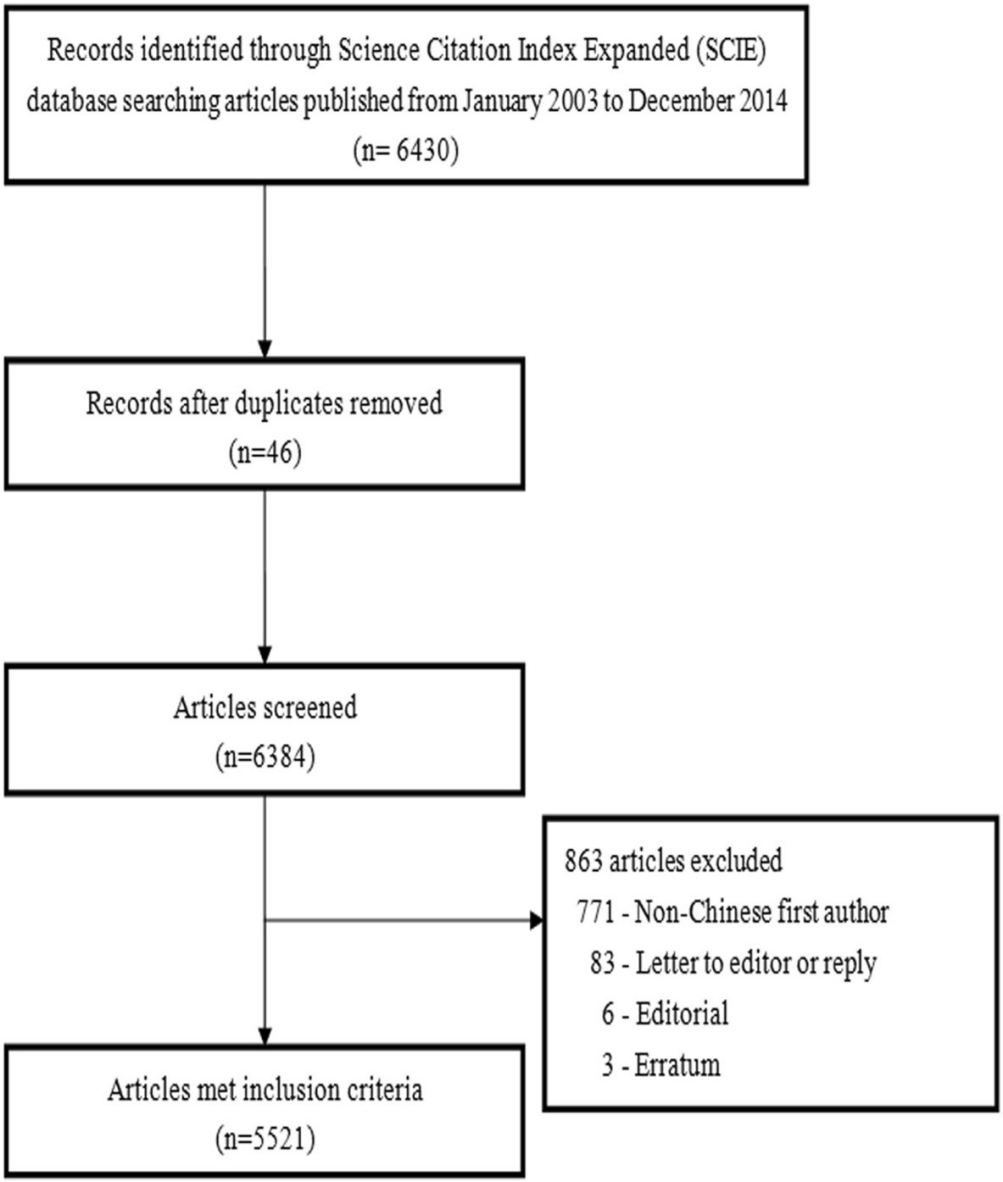
Flow chart for study selection

**Fig. 2:**
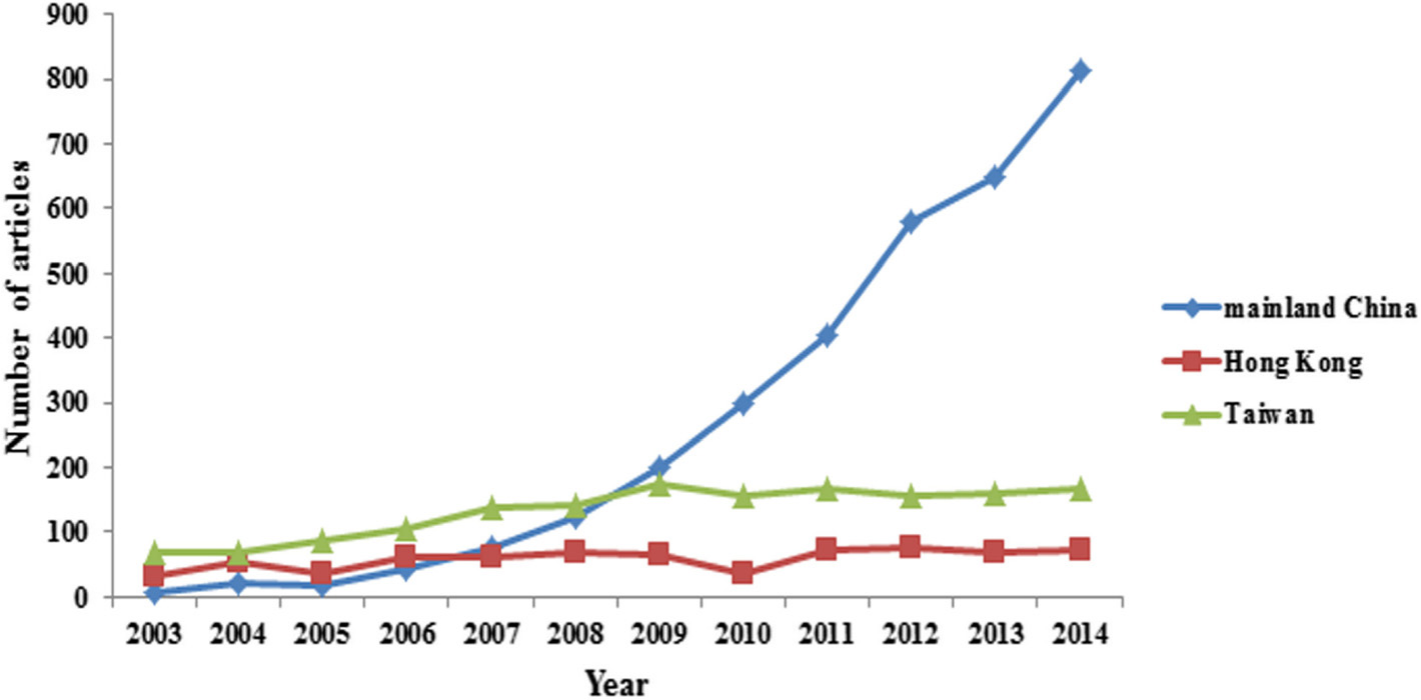
Annual number of articles in the 63 orthopedics journals written by authors from mainland China, Hong Kong, and Taiwan (2003–2014)

**Fig. 3:**
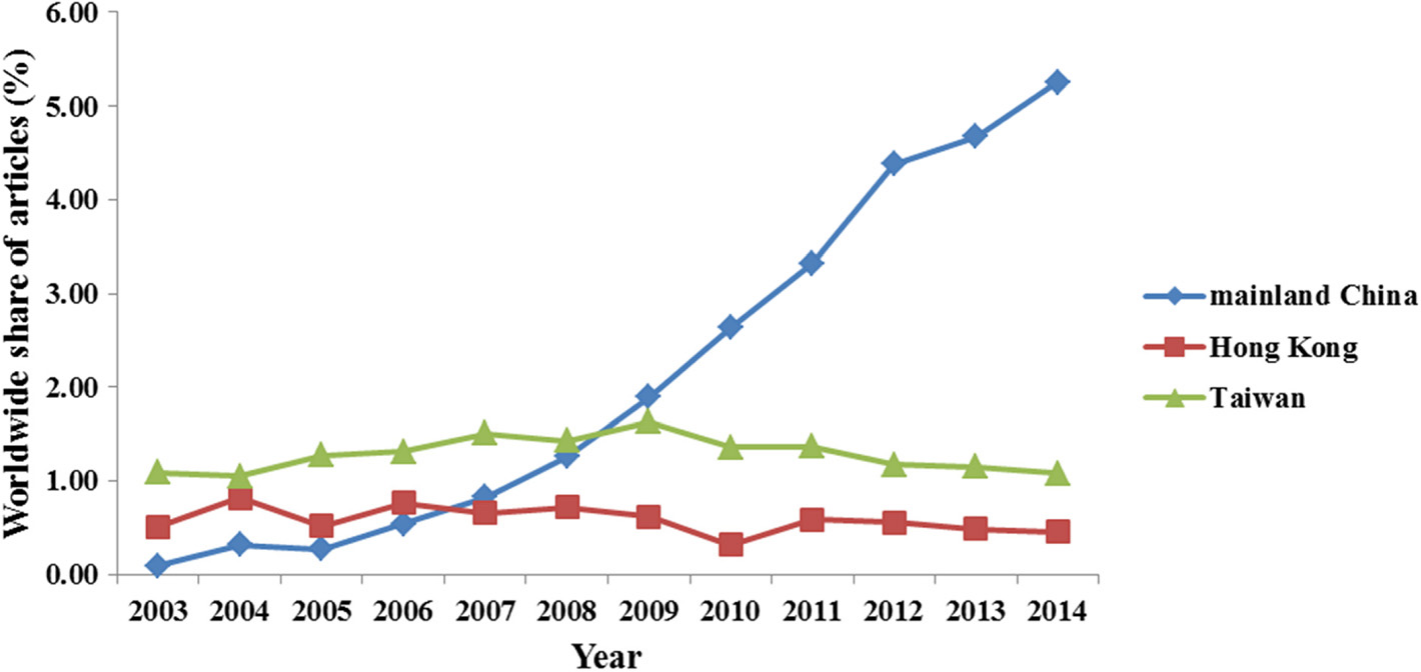
Annual worldwide share of articles in the 63 orthopedics journals written by authors from mainland China, Hong Kong, and Taiwan (2003–2014)

Of the 123,317 published orthopedics articles, 5521 (4.48 %) were from these three regions (Fig. 1), including mainland China (3235/5521, 58.6 %), Hong Kong (700/5521, 12.7 %), and Taiwan (1586/5521, 28.7 %). The annual number of articles from the three regions increased significantly from 2003 to 2014 (mainland China 6 to 813, *R*^2^ = 0.894, *P* < 0.0001; Hong Kong 32 to 71, *R*^2^ = 0.430, *P* = 0.022; Taiwan: 68 to 168, *R*^2^ = 0806, *P* < 0.0001; Fig. 2). The annual number of articles from mainland China has exceeded that from Hong Kong since 2007 and surpassed that from Taiwan in 2009. During the past decade, the number of articles from Taiwan and Hong Kong remained approximately 150 and below 80, respectively. The worldwide share of articles from mainland China increased significantly over time (*R*^2^ = 0.941, *P* < 0.0001), but this was not the case for articles from Taiwan (*R*^2^ = 0.018, *P* = 0.987) and Hong Kong (*R*^2^ = 0.224, *P* = 0.115; Fig. 3).

### Clinical trials, randomized controlled trials, and case reports

**Fig. 4:**
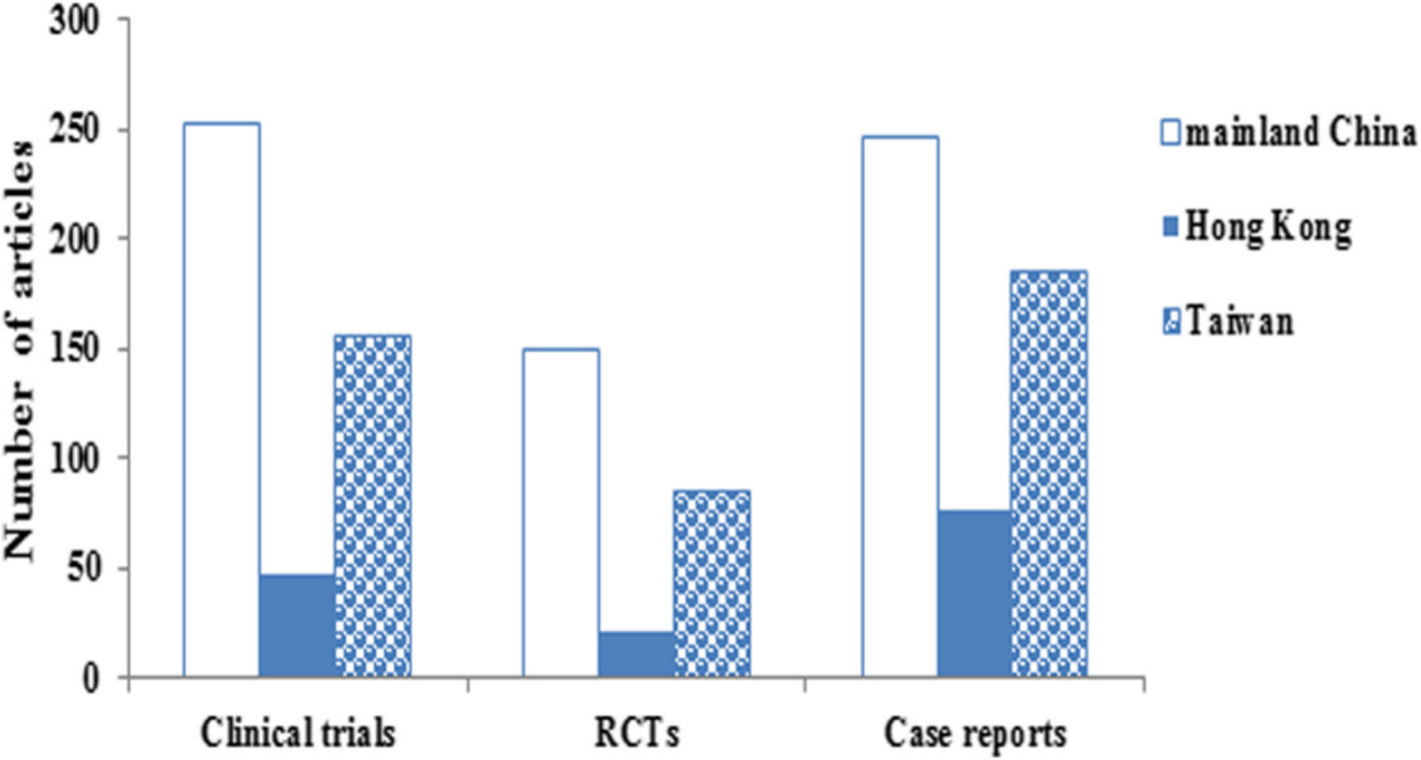
Number of clinical trials, randomized controlled trials (RCTs), and case reports published by authors from mainland China, Hong Kong, and Taiwan (2003–2014)

The total quantity of published RCTs differed significantly among the three regions (*P* = 0.015). The number of RCTs published per year from mainland China has led the three regions since 2010, and even surpassed the combined number of RCTs published per year from Hong Kong and Taiwan. The number of published clinical trials from mainland China was more than those from Hong Kong or Taiwan (mainland China vs. Hong Kong, *P* = 0.003; mainland China vs. Taiwan, *P* = 0.137; Hong Kong vs. Taiwan, *P* = 0.009). Researchers from mainland China published 247 case reports between 2003 and 2014, which far exceeded those from Taiwan (*n* = 186, *P* = 0.02) and Hong Kong (*n* = 76, *P* < 0.001). The number of case reports from Taiwan was significantly higher than that from Hong Kong (*P* = 0.001) (Fig. 4).

### Impact factors

In the current study, the 63 orthopedics journals had IFs based on the JCR 2014 [8]. The accumulated IFs of the articles from mainland China were much higher than those from Taiwan and Hong Kong (6197.33 vs. 3521.78 vs. 1871.46, all *P* values were less than 0.001). However, the average IFs of the articles from mainland China were much lower than those from Hong Kong and Taiwan (2.20 vs. 2.39 vs. 2.43, *P* < 0.001). Hong Kong and Taiwan did not differ significantly in terms of average IFs of articles (*P* > 0.05) (Table 2).

**Table 2:** The accumulated and average impact factors of articles from mainland China, Hong Kong, and Taiwan from 2003 to 2014

Year	Accumulated impact factors			Average impact factors		
	Mainland China	Hong Kong	Taiwan	Mainland China	Hong Kong	Taiwan
2003	15.73	85.61	189.06	2.62	2.31	2.74
2004	47.56	152.80	185.20	2.16	2.55	2.44
2005	79.01	111.42	233.52	2.55	2.42	2.51
2006	111.36	160.31	263.15	2.42	2.39	2.63
2007	183.61	161.19	337.02	2.38	2.52	2.79
2008	281.90	143.59	288.63	2.39	2.28	2.43
2009	389.48	194.18	356.23	2.36	2.31	2.33
2010	582.11	104.97	352.96	2.26	2.33	2.43
2011	760.61	210.77	384.26	2.26	2.45	2.43
2012	1089.37	208.48	313.43	2.23	2.32	2.37
2013	1205.97	150.84	307.44	2.13	2.36	2.14
2014	1450.62	187.30	310.87	2.07	2.40	2.27
Total	6197.33	1871.46	3521.78	2.20	2.39	2.43

### Citations of published articles

Orthopedics articles from mainland China were cited the highest number of times (18723), followed by the Taiwan (15760) and Hong Kong (8346). These differences among the three regions were significant (*P* = 0.007). A higher average per article citation was exhibited in the articles from Hong Kong (11.92) (8346 citations/700 articles) compared to those from Taiwan (9.94) (15760 citations/1586 articles) and mainland China (5.79) (18723 citations/3235 articles). This difference was statistically significant (*P* = 0.027) (Table 3).

**Table 3:** Total citations and average citations per article from mainland China, Hong Kong, and Taiwan from 2003 to 2014

	Total citations			Average citations		
Year	Mainland China	Hong Kong	Taiwan	Mainland China	Hong Kong	Taiwan
2003	203	635	1461	33.83	19.84	21.49
2004	596	990	1416	28.38	18.33	20.52
2005	868	560	1760	45.68	15.56	20.00
2006	1336	1073	2174	31.07	17.59	20.70
2007	1273	1213	1780	16.97	20.22	12.99
2008	1802	902	1653	14.65	12.89	11.81
2009	2075	946	1907	10.32	14.33	11.02
2010	2692	511	1281	8.97	14.19	8.26
2011	2475	685	1022	6.11	9.51	6.12
2012	2692	536	734	4.64	7.24	4.71
2013	1995	187	461	3.07	2.75	2.88
2014	716	108	111	0.88	1.52	0.66
Total	18723	8346	15760	5.79	11.92	9.94

### Articles in the top 10 high IF orthopedics journals

A total of 1147 articles from the three regions were published in the top 10 high IF orthopedics journals. Among them, 20.84 % (239/1147) were in the top 3 journals, including *Journal of Bone and Joint Surgery American Volume*, *American Journal of Sports Medicine*, and *Osteoarthritis and Cartilage*. Researchers from mainland China published 518 articles (45.16 %) in the top 10 high IF orthopedics journals, those from Hong Kong published 193 articles (16.82 %), and those from Taiwan published 436 articles (38.02 %) (mainland China vs. Hong Kong, *P* = 0.003; mainland China vs. Taiwan, *P* = 0.456; Hong Kong vs. Taiwan, *P* = 0.017) (Table 4).

**Table 4:** Articles published in the top 10 high IF orthopedics journals by researchers from mainland China, Hong Kong, and Taiwan from 2003 to 2014

Rank	Journal	2014 IF	Mainland China	Hong Kong	Taiwan	Total
1	J Bone Joint Surg Am	5.28	34	11	49	94
2	Am J Sport Med	4.362	28	13	23	64
3	Osteoarthritis Cartilage	4.165	46	6	29	81
4	J Bone Joint Srug Br	3.309	35	10	30	75
5	Arthroscopy	3.206	60	31	59	150
6	Knee Surg Sport Tr A	3.053	82	27	26	135
7	J Orthop Res	2.986	87	35	103	225
8	Acta Orthopaedica	2.771	11	3	11	25
9	Clin Orthop Relat R	2.765	68	30	56	154
10	Journal of Arthroplasty	2.666	67	27	50	144
Total			518	193	436	1147

### Top 10 popular orthopedics journals

The journals that published the most articles written by orthopedist from the three regions are listed in Table 5. Over 12 years, *Spine* ranked the first in mainland China, Hong Kong, and Taiwan. Besides *Spine*, *Injury* and *Journal of Orthopaedic Research* also appeared in the list of most popular journals for all three regions.

**Table 5:** Top 10 orthopedics journals publishing the most articles written by authors from mainland China, Hong Kong, and Taiwan from 2003 to 2014

Rank	Mainland China	IF	*N*	Hong Kong	IF	*N*	Taiwan	IF	*N*
1	Spine	2.297	327	Spine	2.297	83	Spine	2.297	127
2	ESJ	2.066	267	JOR	2.986	35	JOR	2.986	103
3	IO	2.11	254	ARTH	3.206	31	Injury	2.137	93
4	ORTH	0.962	180	CORR	2.765	30	ARTH	3.206	59
5	AOTS	1.597	151	Injury	2.137	29	CORR	2.765	56
6	Injury	2.137	137	KSSTA	3.053	27	JOA	2.666	56
7	JSDT	2.202	134	JOA	2.666	27	ORTH	0.962	55
8	JOR	2.986	87	IO	2.11	27	AOTS	1.31	53
9	KSSTA	3.053	82	FAI	1.506	24	BMD	1.717	53
10	JHSA	1.667	80	TSJ	2.426	17	JBJSA	5.28	49

*Abbreviations*: *ESJ* Eur Spine J, *IO* Int Orthop, *ORTH* Orthopedics, *AOTS* Arch Orthop Traum Su, *JSDT* J Spinal Disord Tech, *JOR* J Orthop Res, *KSSTA* Knee Surg Sport Tr A, *JHSA* J Hand Surg Am, *ARTH* Arthroscopy, *CORR* Clin Orthop Relat R, *JOA* J Arthroplasty, *FAI* Foot Ankle Int, *TSJ* Spine J, *BMD* BMC Musculoskel Dis, *JBJSA* J Bone Joint Surg Am

### Most prolific and highly cited authors

Researchers were ranked based on their number of articles and citations (Tables 6 and 7). Among the top 10 most published authors, 4 were from Taiwan, 3 were from mainland China, and 3 were from Hong Kong. The number of publications for the top 10 authors ranged from 84 articles (1st place) to 64 articles (10th place). Among the citation ranking of the top 10 authors, 4 were from Taiwan, 4 were from Hong Kong, and 2 were from mainland China. The number of citations for the top 10 authors ranged from 1625 (1st) to 810 (10th). Qiu Y, from mainland China, was the most prolific author, publishing 84 articles during the study period, while Wang CJ, from Taiwan, was the most-cited author (1625 citations). Seven of the top 10 prolific authors had citations within the top 10, including Luk KDK (Hong Kong), Chen Wen-Jong (Taiwan), Wang CJ (Taiwan), Chen Wen-Jer (Taiwan), Tang JB (mainland China), Cheung KMC (Hong Kong), and Chen TH (Taiwan). The orthopedist outside of the top 10 most published authors was not inevitably low in the citation rates of articles. For instance, the 60 articles by Cheng JCY (Hong Kong) ranked him in 12th place overall, but his articles ranked 6th in total number of citations (*n* = 1048). Qin L (Hong Kong) ranked 18th (*n* = 46) in number of articles and 9th (*n* = 878) in total number of citations.

**Table 6:** Top 10 authors, according to the number of articles from 2003 to 2014

Rank	Author	No. of articles	No. of citations	Average citation	*H*-index	Region
1	Qiu Yong	84	536	6.38	12	ML
2	Luk Keith D.K.	82	1274	15.54	20	HK
3	Chen Wen-Jong	76	1134	14.92	22	TW
4	Lui Tun Hing	75	362	4.83	10	HK
5	Wang Ching-Jen	71	1625	22.89	22	TW
6	Chen Wen-Jer	70	1074	15.34	21	TW
7	Tang Jingbo	67	967	14.43	18	ML
8	Yuan Wen	65	327	5.03	10	ML
9	Cheung Kenneth M. C.	65	1136	17.48	19	HK
10	Chen Tain-Hsiung	64	810	12.66	15	TW

*Abbreviations*: *ML* mainland China, *HK* Hong Kong, *TW* Taiwan

**Table 7:** Top 10 authors, according to the number of citations from 2003 to 2014

Rank	Author	No. of citations	No. of articles	Average citation	*H*-index	Region
1	Wang Ching-Jen	1625	71	22.89	22	TW
2	Luk Keith D.K.	1274	82	15.54	20	HK
3	Cheung Kenneth M.C.	1136	65	17.48	19	HK
4	Chen Wen-Jong	1134	76	14.92	22	TW
5	Chen Wen-Jer	1074	70	15.34	21	TW
6	Cheng Jack C.Y.	1048	60	17.47	18	HK
7	Tang Jingbo	967	67	14.43	18	ML
8	Dai Li-Yang	908	57	15.93	19	ML
9	Qin Lin	878	46	19.09	17	HK
10	Chen Tain-Hsiung	810	64	12.66	15	TW

*Abbreviations*: *TW* Taiwan, *HK* Hong Kong, *ML* mainland China

### Most prolific and highly cited institutions

Among the top 10 most prolific institutions, 4 were in mainland China, 4 were in Taiwan, and 2 were in Hong Kong (Tables 8 and 9). The number of publications by the top 10 institutions ranged from 421 articles (1st place) to 101 articles (10th place). The number of citations by the top 10 institutions ranged from 5500 (1st place) to 931 (10th place). Chang Gung Memorial Hospital was the most prolific institutional source of orthopedics articles, and also ranked first in the total number of citations. Eight of the top 10 prolific institutions had citations within the top 10, except for Changzheng Hospital and West China Hospital. However, the centers not in the top 10 most prolific institutions were not certainly low in the total citations of articles. For example, Xinhua Hospital affiliated to Shanghai Jiao Tong University School of Medicine ranked 18th in number of published articles (*n* = 70) and 8th in total number of citations (*n* = 976). Kaohsiung Medical University ranked 14th in number of published articles (*n* = 82) and 9th in total number of citations (*n* = 966).

**Table 8:** Top 10 institutions, according to the number of articles from 2003 to 2014

Rank	Institution	No. of articles	No. of citations	Average citations	*H*-index	Region
1	Chang Gung Mem Hosp	421	5500	13.06	34	TW
2	Prince Wales Hosp	244	3192	13.08	31	HK
3	Vet Gen Hosp	226	2299	10.17	26	TW
4	Natl Taiwan Univ Hosp	185	2048	11.07	23	TW
5	Queen Mary Hosp	153	1795	11.73	24	HK
6	Natl Cheng Kung Univ	148	1419	9.59	18	TW
7	Changzheng Hosp	146	780	5.34	16	ML
8	Shanghai Peoples Hosp 6	131	931	7.11	16	ML
9	West China Hosp	130	735	5.65	12	ML
10	Peking Univ Hosp 3	101	992	9.82	16	ML

*Abbreviations*: *TW* Taiwan, *HK* Hong Kong, *ML* mainland China

**Table 9:** Top 10 institutions, according to number of citations from 2003 to 2014

Rank	Institution	No. of citations	No. of articles	Average citations	*H*-index	Region
1	Chang Gung Mem Hosp	5500	421	13.06	34	TW
2	Prince Wales Hosp	3192	244	13.08	31	HK
3	Vet Gen Hosp	2299	226	10.17	26	TW
4	Natl Taiwan Univ Hosp	2048	185	11.07	23	TW
5	Queen Mary Hosp	1795	153	11.73	24	HK
6	Natl Cheng Kung Univ	1419	148	9.59	18	TW
7	Peking Univ Hosp 3	992	101	9.82	16	ML
8	Xinhua Hosp	976	70	13.94	20	ML
9	Kaohsiung Med Univ	966	82	11.78	16	TW
10	Shanghai Peoples Hosp 6	931	131	7.11	16	ML

*Abbreviations*: *TW* Taiwan, *HK* Hong Kong, *ML* mainland China

## Discussion

To the best of our knowledge, this is the first study to systematically analyze the quantity and quality of literature regarding orthopedics diseases from Chinese authors in the three regions (mainland China, Hong Kong, and Taiwan). Our study demonstrated that China had a consistent improvement in the field of orthopedics, and the numbers of articles published every year have increased markedly from 2003 to 2014. Worldwide there was also a persistent increase in the number of orthopedics publications during the past 12 years. Table 1 could give a general picture of the worldwide productivity in orthopedics research. It was very difficult to discuss about all countries mentioned in the Table 1. Therefore, we only focused on the traditional scientific powers and rapidly emerging scientific powers, which made a major contribution to the field of orthopedics. There is no doubt that the total and annual numbers of orthopedics publications from USA were the highest in the world. In the past 12 years, the annual number of published articles increased steadily in highly developed countries including the UK, Japan, Germany, and Canada. Of note, there was a dramatic increase in the annual number of published articles in China and South Korea. On the other hand, publications per million (the ratio of the number of publications to the population of a country) was one of the parameters used in the measurement of scientific productivity of a community. Small highly developed countries, such as Denmark, Finland, Sweden, and the Netherlands, all ranked in the top in respect to orthopedics publications per million, and outperformed larger highly developed counterparts including USA, Germany, and Japan [9–11]. Given the relative size of the populations, China lagged far behind the most productive countries in the field of orthopedics.

As it was a British Colony since the middle of the 19th century, Hong Kong has a long history of internationalization and a high degree of modern science and education level. Taiwan was one of the famous Newly Industrial Economics countries from the 1970s, whose advanced scientific and academic systems mixed features of China and America. For many years, scientific and medical research from Hong Kong and Taiwan has a world-class performance, and has been more advanced than that from mainland China. In fact, researchers from these two regions have contributed some of the best scientific articles on orthopedics. On the other hand, orthopedics in mainland China has made a great progress since the beginning of the 21st century. Its rapid growth has occurred in the recent 12 years due to the implementation of reform and open policy, and with the opening of China’s doors to the international community. In accordance with previous studies in other disciplines [12, 13], our results clearly demonstrated that scientific publication by mainland China investigators in international orthopedics periodicals increased dramatically during this period. Specifically, the annual number and worldwide share of articles from mainland China has exceeded that from Hong Kong since 2007 and surpassed that from Taiwan in 2009. The number of articles from Hong Kong increased rapidly from 2003 to 2006, and this number has plateaued since 2006. An increasing trend in the number of articles from Taiwan was found between 2003 and 2009 and remained steady at the end of the study period.

Mainland China had the largest number of clinical trials, RCTs, and case reports during the past 12 years. Historically, investigators from Taiwan have contributed a great number of clinical trials and RCTs in the field of orthopedics. However, the annual number of such studies from researchers in mainland China has been ahead of the three regions since 2010. In addition, the numbers of published case reports from mainland China also far exceeded those from Hong Kong and Taiwan. There are several reasons that clinical research in mainland China has proceeded at a fast speed. First, China is a large country, with the largest population in the world and a vast number of patients suffering from orthopedics diseases. Second, people in mainland China may be more compliant towards engagement in clinical research protocols. Third, clinical trials in mainland China are much less costly to carry out [14]. Fourth, controlled clinical studies have been an important research direction of Chinese orthopedists in recent years [15].

The annual total IFs of articles originating in China has increased significantly over the past 12 years, and has surpassed Taiwan and Hong Kong since 2009. However, the average IFs of articles from mainland China still lags behind those from Taiwan and Hong Kong. It should also be mentioned that IFs are not the only or optimal parameters for determining the quality of articles [16]. The number of times an article has been cited represents the degree of its influence on other publications and was adopted in this study as an indicator of the impact and quality of articles. Articles from mainland China were cited most, those from Taiwan were cited second-most, and those from Hong Kong were cited the least. However, the average citations per article of publications from mainland China were markedly lower than those from Taiwan and Hong Kong. Mainland China witnessed a rapid increase in the number of published orthopedics articles, but its total and average citations per article were small. This phenomenon could be explained by two reasons. On one hand, mainland China published 2042 articles in the most recent 3 years, accounting for 63.12 % (2042/3235) of the total number of articles published during 2003 to 2014. Obviously, the older an article is, the more likelihood it has of being cited. On the other hand, this may reflect the fact that quality of articles from mainland China needs to be improved significantly.

As far as the top 10 high IF orthopedics journals are concerned, researchers from mainland China (518 articles) and Taiwan (436 articles) published more papers than those from Hong Kong (193 articles). In the present study, *Spine*, *Injury*, and *Journal of Orthopaedic Research* were found to be the most popular journals for authors from mainland China, Hong Kong, and Taiwan and were in the list of the top 10 most popular journals in all three regions. These journals are the oldest publications for the specialty of orthopedics, and have an established and proud history of publishing top-class scientific articles in the field. During the past 12 years, 537 articles from Chinese authors were published in *Spine*, 259 articles were published in *Injury*, and 225 articles were published in *Journal of Orthopaedic Research*. Recently, the introduction of a Chinese-language edition of high-level journals may have increased authors’ interest in publishing articles in these journals, which have certainly promoted the development of orthopedics research in China and contributed to China taking its place in the field of orthopedics.

An analysis of prolific authors in orthopedics showed that Taiwan had 4 most prolific authors, and mainland China and Hong Kong each had 3 most prolific authors. Furthermore, Taiwan and Hong Kong both had 4 most-cited orthopedics researchers, and mainland China had 2 most-cited orthopedics researchers. An analysis of the top 10 research institutions found that the mainland China and Taiwan each had the 4 top research institutions for orthopedics, while Hong Kong had 2 top research institutions. However, the top 6 research institutions all came from Taiwan and Hong Kong. Except for West China Hospital, three of four top research institutions from mainland China were located in the southeast coastal region that was considered as the most developed area of China. It is objective fact that the development of medicine in eastern and western China exist the regional disparity. In Taiwan, the Chang Gung Memorial Hospital has been considered a “powerhouse”, which published 421 articles with 5500 citations, accounting for 26.5 % of Taiwan’s publications.

Publications in the medical field that reach an international audience mainly depend on excellent research and English fluency. This has given a major advantage to “English-speaking” or developed regions. Hong Kong, as a previous British colony, has the superiority of having a strong foundation in written English. In contrast, Taiwan is generally better known for having one of the best research environments in East Asia, owing to its socioeconomic development. Thus, academic institutions from Hong Kong and Taiwan have been able to publish more high-quality papers in orthopedics science. In recent years, mainland China has narrowed the gap, and has even overtaken the Hong Kong and Taiwan, at least in terms of the number of articles published in international orthopedics journals. However, the most prolific authors and top research institutions from mainland China still lag behind those from Hong Kong and Taiwan in terms of citations per article. Our study indicates that mainland China has come a long way and still has some distance to achieve the academic productivity of Hong Kong and Taiwan.

Several factors favor mainland China’s immense growth in orthopedics research. First, as the recent advances in China’s economy, the public are beginning to raise awareness of bone and joint disease [17]. Many new institutions and hospitals in mainland China have started basic and clinical research in the orthopedics fields [18]. Second, international collaborations may help Chinese orthopedics investigators to improve their clinical orthopedics practice and research capabilities, and make it possible for overseas scholars to understand the development of orthopedics science in mainland China. Third, in recent years, Chinese government authorities have begun to advocate for the use of IFs and citations when evaluating the performance of individual scientists. This has now been regarded as an important indicators used to measure their scientific contributions, which is closely related to academic status, income, funding, and other important benefits [19]. Subsequently, scientists have endeavored to publish their research in SCIE journals.

This study has limitations that should be highlighted. First, only the 63 orthopedics journals covered by the SCIE database were analyzed. Some orthopedics articles are published in general medical journals and were not included in this study. Second, the SCIE database is an English-language resource and has a publication bias. Many articles published in the journals from non-English speaking countries that were not indexed in this database. Thus, the contribution of non-English-language publications may have been underestimated. Third, the orthopedics journals were selected from the orthopedics category of SCIE database, and the IFs were evaluated by JCR 2014. In the past 12 years, the included journals and IF of the journals have changed year by year.

## Conclusions

In conclusion, this study provides a novel overview of current Chinese orthopedics research. The annual number of articles published from mainland China has increased markedly during the past 12 years, particularly since 2007. Concurrently, the number of publications from Hong Kong and Taiwan is growing at a slow and steady rate. Publications from mainland China had the highest accumulated IF and total citations of articles. Publications from Taiwan had the highest average citations per article, and publications from Hong Kong had the highest average IFs. Taking into consideration the relative size of the populations, it should be emphasized that mainland China still has a long way to go to achieve the academic performance of Hong Kong and Taiwan.

## Abbreviations

IFs, impact factors; RCTs, randomized controlled trials; SCIE, science citation index expanded
